# Soil organic matter turnover rates increase to match increased inputs in grazed grasslands

**DOI:** 10.1007/s10533-021-00838-z

**Published:** 2021-08-27

**Authors:** Shane W. Stoner, Alison M. Hoyt, Susan Trumbore, Carlos A. Sierra, Marion Schrumpf, Sebastian Doetterl, W. Troy Baisden, Louis A. Schipper

**Affiliations:** 1 Max Planck Institute for Biogeochemistry, Jena, Germany; 2 Department of Environmental Systems Science, ETH Zürich, Zurich, Switzerland; 3 Lawrence Berkeley National Laboratory, Berkeley, CA USA; 4 Environmental Research Institute, University of Waikato, Hamilton, Aotearoa New Zealand; 5 Te Pūnaha Matatini Centre of Research Excellence, Auckland, New Zealand

**Keywords:** Radiocarbon, Soil carbon, Soil modeling, Carbon sequestration, Transit time, SoilR

## Abstract

Managed grasslands have the potential to store carbon (C) and partially mitigate climate change. However, it remains difficult to predict potential C storage under a given soil or management practice. To study C storage dynamics due to long-term (1952–2009) phosphorus (P) fertilizer and irrigation treatments in New Zealand grasslands, we measured radiocarbon (^14^C) in archived soil along with observed changes in C stocks to constrain a compartmental soil model. Productivity increases from P application and irrigation in these trials resulted in very similar C accumulation rates between 1959 and 2009. The ∆^14^C changes over the same time period were similar in plots that were both irrigated and fertilized, and only differed in a non-irrigated fertilized plot. Model results indicated that decomposition rates of fast cycling C (0.1 to 0.2 year^−1^) increased to nearly offset increases in inputs. With increasing P fertilization, decomposition rates also increased in the slow pool (0.005 to 0.008 year^−1^). Our findings show sustained, significant (i.e. greater than 4 per mille) increases in C stocks regardless of treatment or inputs. As the majority of fresh inputs remain in the soil for less than 10 years, these long term increases reflect dynamics of the slow pool. Additionally, frequent irrigation was associated with reduced stocks and increased decomposition of fresh plant material. Rates of C gain and decay highlight trade-offs between productivity, nutrient availability, and soil C sequestration as a climate change mitigation strategy.

**Supplementary Information:**

The online version contains supplementary material available at 10.1007/s10533-021-00838-z.

## Introduction

Globally, grassland soils store approximately 340 Pg carbon (C) (Conant et al. 2017), comprising about 23% of the global soil C stock to 1 m depth (Batjes 2016; FAO 2017) and covering roughly one third of global land surface (Rutledge et al. 2017; McNally et al. 2015). Grasslands may therefore offer large potential for sequestering atmospheric CO_2_ in soil to mitigate anthropogenic climate change (Reid et al. 2004; Neely et al. 2009; Minasny et al. 2017). Additionally, roughly 70% of global grasslands are managed (Conant et al. 2017), placing significant control of global soil C stocks into the hands of land managers. Management strategies for increasing soil C sequestration typically rely on increasing productivity (Conant et al. 2001, 2017), a potential win–win approach. However, practices that increase productivity in grasslands such as fertilization and irrigation in drier regions could also affect SOC decomposition rates since greater nutrient availability and longer periods of sufficient wetness in soil can stimulate heterotrophic microorganisms, leading to potential trade-offs. Thus, understanding the interactions between increasing inputs and decomposition is essential for informing management decisions to increase soil C stocks and extend the time this C is sequestered in soils (McSherry and Ritchie 2013; Conant et al. 2017; Guo and Macdonald 2006).

Livestock grazing is a dominant management regime for dryland grasslands globally (FAO 2019). Grazed pasture often occupies areas where arable crops are not feasible due to scarcity in water and available nutrients, primarily phosphorus (P) and nitrogen (N). For example, irrigation and fertilization with P is a common practice on grass-clover pastures in New Zealand, boosting biotic N fixation and overall productivity up to three-fold (Ball 1969; Schipper et al. 2013).

In most managed grasslands, such increases in productivity result in greater soil C stocks (Conant et al. 2017). The relationship between increased inputs and subsequent storage varies depending on numerous factors (Six et al. 2002; West and Six 2007). Indeed, many experiments showed (Campbell et al. 1991; Reicosky et al. 2002; Douglas et al. 2020) and early models predicted (Jenny 1941; Andren and Katterer 1997; Paustian et al. 1997) C content to increase at a nearly linear rate with increased inputs. However, more recent work has suggested limits to sequestration and introduced the concept of a potential maximum soil C sequestration capacity (C saturation) that is related to soil mineralogy (Six et al. 2002; Stewart et al. 2007; Campbell and Paustian 2015; Dexter et al. 2008) or microbial activity and community composition (Blagodatskaya and Kuzyakov 2008).

Conversely, intensification of pasture management regimes, e.g. irrigation and fertilization, that increase productivity may also stimulate soil organic matter (SOM) decomposition. For example, large releases of respired soil C are well-established following rewetting of dry soil (Birch 1964; Jarvis et al. 2007; Lado-Monserrat et al. 2014), and baseline respiration tends to be higher in continually wet soils (Fierer and Schimel 2002; Hou et al. 2019). Relieving nutrient limitations through fertilization may either (i) decrease decomposition (negative priming) by preventing so-called “nutrient mining” from more stable SOM fractions (Kuzyakov and Domanski 2000), or (ii) increase decomposition (positive priming) of old C or by increasing labile substrates to overcome barriers to decomposition (Luo et al. 2016). Sanderman et al. (2017) showed that productive pastures with high C stocks experienced both accumulation of microbial necromass, considered to be a primary component of stable SOM (Cordova et al. 2018), as well as accelerated SOM turnover. Given co-limitations to microbial activity, broadening our understanding of cumulative influences controlling trade-offs between plant productivity and SOM stability over time requires further investigation in grasslands.

Mitigating climate change through increases in SOM storage requires that management decisions be informed by land use impacts on complex SOM stabilization mechanisms. Losses of old SOM may be compensated by increased inputs, but in the absence of stabilizing processes these inputs are short-lived and management must therefore be maintained indefinitely to retain elevated SOM stocks. Adopting management that increases the time C stays in soils, i.e. its transit time, provides greater potential for soils to retain atmospheric CO_2_ on decadal timescales or longer (Schlesinger and Amundson 2019; Amundson and Biardeau 2018; Sierra et al. 2021).

To investigate the long-term impacts of management intensification through irrigation and P fertilization, we investigated the long-term research site at the Winchmore Irrigation Research Station on New Zealand’s South Island near Canterbury (McDowell et al. 2021). In 1948, historically low-producing browntop pasture was scraped (removing the top 1–2 cm) to create irrigation border dykes, then converted to plots with intensive moisture and fertility management intended to boost plant growth. Thus, plots have undergone disturbance, recovery, and increases in plant productivity and C inputs. Monitoring over the 60 years of treatment showed that P fertilization significantly increased pasture productivity but, though all treatments continually accumulated C, application rates had no effect on SOM stock accumulation rates (Schipper et al. 2013; Condron et al. 2014). Irrigation also enhanced aboveground production but heavily irrigated soils maintained about 20% lower SOM stock compared with less-irrigated and dryland pastures. A lack of greater increase in C stocks despite greater inputs in the Winchmore fertilization and irrigation trials implies that output fluxes must have also increased to off-set inputs.

Decadal soil radiocarbon (^14^C) trends can offer further insights into the cumulative effects of long-term management on soil processes. Fortunately, a nearly annual sampling of Winchmore soils has been archived from 1959 to 2009, enabling long-term ^14^C measurements. Moreover, the impeccable timing of the Winchmore archive captures the complete ^14^C “bomb” pulse. Atmospheric testing of thermonuclear weapons in the 1950s and 1960s produced a sharp increase in global atmospheric ∆^14^C (Fig. 2) that can be modeled as a tracer of plant C inputs and can constrain C dynamics on decadal scales (Baisden et al. 2002; Schuur et al. 2016). Furthermore, robust ^14^C time series data in a changing system may shed light on critical modeling assumptions (Baisden et al. 2013).

Addition of ∆^14^C measurements allows more quantitative assessment of changes in C dynamics with management. Specifically, published studies of the Winchmore and Waite trials found increases in productivity did not increase rates of SOM storage and suggest that decomposition rates increased to offset higher inputs (Schipper et al. 2013; Sanderman et al. 2017). However, the same observation of bulk C changes could reflect different dynamical responses. For example, in nutrient addition plots, increases in bulk SOM could reflect accumulation of new inputs balanced by simultaneous erosion of older SOM pools via ‘priming’. Alternatively, SOM increases could be explained by decomposition rates of ‘faster’ cycling pools increasing to offset increased inputs, with no change in 'slow' pool dynamics. While the trajectory of SOM stocks in these cases would be the same, the ^14^C signature of the bulk C would reflect the fact that the SOM should be younger for the former case and older for the latter. Using ^14^C together with models allows quantification of C cycling rates for each of the treatments applied, and allows for improved process understanding.

Therefore we measured ^14^C in the archived soils from the Winchmore trials and modeled simultaneous changes in ∆^14^C and C stocks using a two-pool model to resolve how decomposition rates changed in faster- and slower-cycling pools. In order to explain the lack of change in SOM accumulation rates with increased pasture productivity following P application, we hypothesize that P application increased the decomposition rates in both faster and more slowly cycling soil C pools. Carbon loss was then replaced through stimulated plant productivity, resulting in similar stocks but overall a greater fraction of recently fixed C in high P treatments as indicated by ^14^C. Under irrigation manipulation, we expect wet soils with lower C stocks to undergo more rapid decay in the fast pool to maintain lower C stocks.

## Methods

### Site description and experimental design

The Winchmore trials are long-term agricultural research experiments that have been carefully studied, including changes in C stocks, for over 70 years (McDowell et al. 2021; Schipper et al. 2013). Two pasture productivity experiments sharing similar site history were selected for this analysis (Table 1). In 1948, the top 1–2 cm of soil were scraped from the center of pastures toward edges to create border dykes used to contain flood irrigation. During each irrigation event about 100 mm of standing water was applied. All pastures were grazed by sheep, and stocking rates were adjusted to match pasture production (Schipper et al. 2013).

**Table 1:** Adapted from Schipper et al. (2013)

Trial	Irrigation regime^a^	Fertilizer regime (kg ha^−1^ year^−1^)	Aboveground (*θ*_*AG*_ = 0.29) (S.D.)	Belowground (*θ*_*BG*_ = 0.7)^b^	Mean total inputs (Mg ha^−1^ year^−1^)	Mean SOC increase (Mg ha^−1^ year^−1^)^c,d^	Capture efficiency (% inputs stored)
Unfert	15%	–	1.9 (0.53)	1.9	1.9 (0.2)	0.22	11.6
Res. fert	15%	376-0-250^e^	3.8 (1.35)	2.1^f^	2.6 (0.4)	0.23	8.8
High fert	15%	376	4.8 (0.79)	2.3	3.0 (0.2)	0.20	6.7
Dryland	–	250	2.8 (0.80)	2.0	2.2 (0.2)	0.16	7.3
Irr. 10	10%	250	4.0 (0.78)	2.1^f^	2.6 (0.2)	0.15	5.8
Irr. 20	20%	250	4.8 (0.73)	2.1	2.8 (0.2)	0.13	4.6

Mean annual above- and belowground production (in Mg ha^−1^ year^−1^), and mean total inputs after applying *θ* factors to convert from production to assumed inputs. Annual input data is available in Online Appendix Fig. 1. Capture efficiency is calculated as total inputs divided by annual increase in SOC. Soil moisture of 10% represents approximately permanent wilting point, and 20% is approximately 50% field capacity (White et al. 2008)

^a^Indicates soil moisture reached before triggering an irrigation event. 10% soil moisture corresponds to permanent wilting point, 20% soil moisture is roughly 50% of field capacity

^b^No significant differences among trials (Irrigation LSD: 0.36, Fertilizer LSD: 0.99) from Scott et al. (2012)

^c^No significant differences between trials (Irrigation SEM: 0.027, Fertilizer SEM: 0.019) from Schipper et al. (2013). SD calculated from available data for all years (Online Appendix Fig. 1)

^d^To depth of 7.5 cm, assuming bulk density of 1.14 g cm^−3^ from Schipper et al. (2013)

^e^The residual fertilizer plot received 376 kg ha^−1^ year^−1^ between 1952 and 1957, then no further fertilizer was added through 1979. In 1980, 860 kg ha^−1^ were applied. From 1981, 250 kg ha^−1^ year^−1^ were applied until the end of the experiment

^d^Irr. 10 and Res. Fert data were not measured, and were thus estimated using mean values

The first analysis focused on irrigation effects on pasture production. Here we analyzed samples from three treatments: (1) the unirrigated control (“Dryland”), which was also scraped free of 1–2 cm topsoil, (2) soil irrigated when moisture content reached 10%, near the permanent wilting point (“Irr. 10”), and (3) soil irrigated when moisture content reached 20%, or roughly 50% of field capacity (“Irr. 20”). All pastures in the irrigation experiment received 250 kg ha^−1^ year^−1^ fertilizer (superphosphate, 7–9% P).

Our second analysis focused on P fertilizer effects on pasture productivity. Trials selected for ∆^14^C analysis included: (1) unfertilized pasture (“Unfert”); (2) a residual fertilizer trial (“Res. Fert”) to which 376 kg superphosphate ha^−1^ year^−1^ was applied until 1959, then P was withheld until 1980, when 860 kg ha^−1^ superphosphate was applied, and pastures received 250 kg superphosphate ha^−1^ year^−1^ through the end of the experiment; and (3) a pasture receiving 376 kg superphosphate ha^−1^ year^−1^ (“High Fert”). All trials in the fertilizer experiment were irrigated when soil moisture reached 15%. Thus, there was no true common control (unirrigated, unfertilized) between experiments, so we focused on treatment effects within each experiment.

### Soil sampling and analysis

Soils were sampled and archived annually from 1959 (10 years post establishment) to 2002 and 2009 for irrigation and fertilizer experiments, respectively. Soil cores were collected in replicate (n = 10) to 7.5 cm depth along the length of each border, bulked, sieved to 2 mm, air-dried and stored at room temperature. Further details of soil sampling are described by Schipper et al. (2013). Total C for all samples were measured by combustion-IR and previously published by Schipper et al. (2013). Radiocarbon analysis was conducted using accelerator mass spectrometry (MICADAS) at the Max Planck Institute for Biogeochemistry in Jena, Germany (Steinhof et al. 2017) and GNS Science in Lower Hutt, New Zealand, for fertilizer and irrigation experiments, respectively (∆^14^C analytical accuracy ± 3–4‰ + spatial variability ± 8‰, data not shown).

### Soil C modeling

#### Model design and inputs

**Fig. 1:**
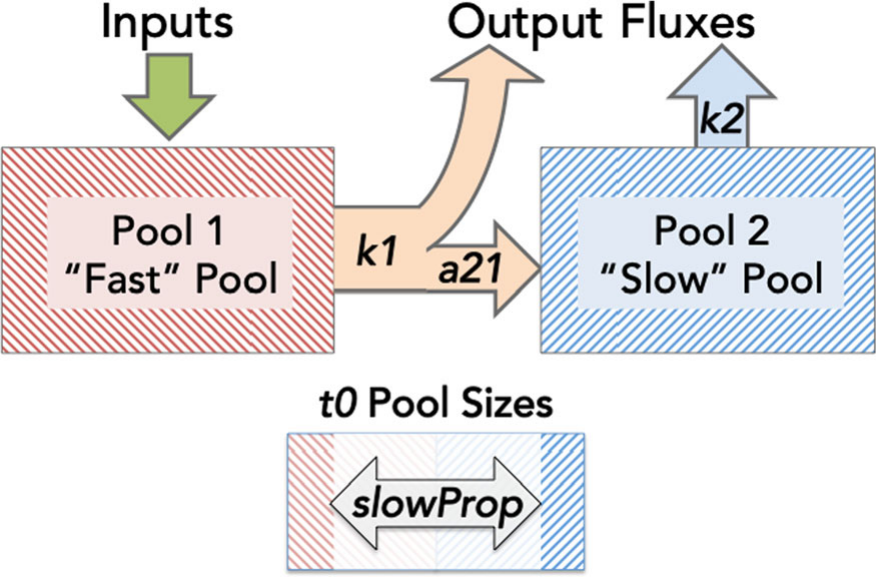
Conceptual diagram of two pool series compartmental model. All inputs enter pool 1. First order decay constants (*k1* and *k2*) control loss from pools 1 and 2, respectively, with transfer coefficient (*a21*) defining the fraction of C leaving pool 1 (*k1* * P1 Stock) and entering pool 2 (k1 * a21 * P1 Stock) annually. Parameter *slowProp* is fit to describe initial (*t0*) distribution of C between pools

Soil C dynamics were estimated using a non-steady-state two-pool series compartmental model (Fig. 1) using the R package SoilR (Sierra et al. 2014; R Core Team 2020). The dynamics of C decay and transfer between pools are described by the equation:

$$ \frac{dC\left( t \right)}{{dt}} = I\left( t \right) + A\left( t \right)C\left( t \right) $$

where ***C****(t)* is a 2 × 1 vector of C stores in each pool at a given time, ***I****(t)* is a time-dependent column vector describing the inputs to each pool, and ***A****(t)* is a 2 × 2 matrix of decomposition and transfer rates between each pool. All inputs enter the fast pool (***I*** = (*inputs(t)*, 0)). For the two-pool series model used here, matrix **A** is given as:

$$ A = \left( {\begin{array}{*{20}c} { - k_{1} } \\ {a_{21} } \\ \end{array} \begin{array}{*{20}c} 0 \\ { - k_{2} } \\ \end{array} } \right) $$

Fitted model parameters include decay constants for each pool (*k1* and *k2*) and a transfer coefficient (*a21*), which describes the rate of C flux from pool 1 (“fast pool”) that is transferred to pool 2 (“slow pool”). A fourth parameter (*slowProp*) was included to fit the proportion of total C in the slow pool at the beginning of the modeled time window to allow flexibility in initial pool sizes, as these were unknown.

Total C inputs were calculated from above- and belowground production data (Online Appendix Fig. 1). Annual pasture production data were used where available (Rickard and Radcliffe 1976; Rickard and McBride 1986) and mean annual production values from each trial were used to fill gaps in each respective time series. Average belowground C production data for these sites were reported by Scott et al. (2012). Contributions from above ground production to total inputs were adjusted by factor *θ*_*AG*_ of 0.29, calculated as a rate of above ground biomass returned to soil by sheep grazing (Jonker et al. 2019). Root production data were reported to 20 cm, and a *θ*_*BG*_ factor of 0.70 was applied to estimate the proportion of roots in the top 0–7.5 cm surface horizon modeled here (Klimek-Kopyra and Rbilas 2018; Liu et al. 2020). Soil C stocks were calculated assuming a bulk density of 1.14 g cm^−3^ (Schipper et al. 2013), typical of a stony loess silt loam (Udic ustochrept). We estimated initial C stocks for each modeled time period by fitting a polynomial regression to the C stock time series to estimate treatment means (Online Appendix Fig. 2). This approach gave equal weight to all C stock data, rather than assigning greater weight to year 1 of each window. Atmospheric ∆^14^C data for the Southern Hemisphere are described by Graven et al. (2017).

#### Modeling windows and initial conditions

We were unable to fit the entire 62-year timespan of the experiments satisfactorily with a single set of parameters. Thus, we split the data into two overlapping time windows, 1958–1992 and 1985–2010, and estimated best-fit parameters for each time window independently. Splitting the time window in this way significantly improved data fitting (e.g. ∆^14^C RMSE of 6.9 vs. 10.46‰, C stock RMSE of 0.96 vs. 2.26 Mg C ha^−1^ for 1958–1992 and 1958–2010, respectively). Windows were selected to (i) ensure strong ^14^C constraint, with a data point at the beginning and end of the modeled window, (ii) ensure roughly equivalent window lengths (34 and 25 years, respectively) and number of ^14^C data points, and (iii) to allow time to elapse before the start of the second window in order to reduce initial management and disturbance effects.

**Fig. 2:**
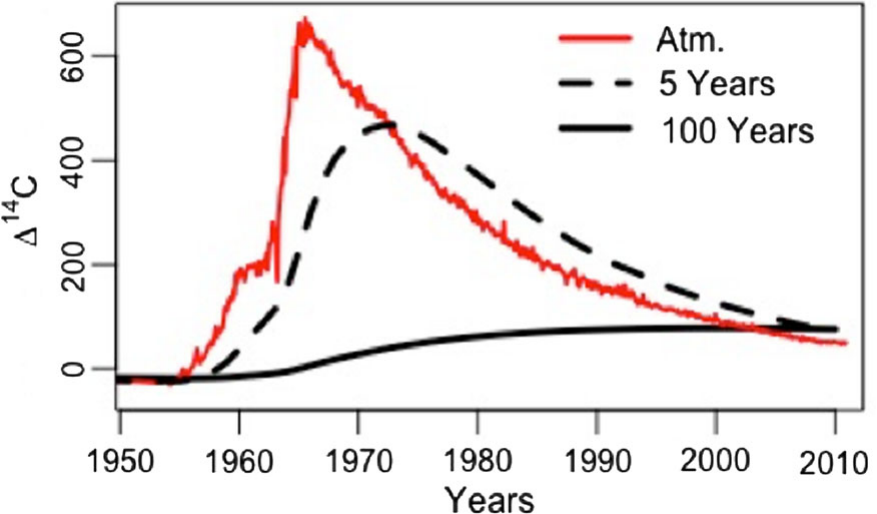
∆^14^C trends for single pools of given turnover times (defined here as the inverse of the decomposition rates, i.e. 1/*k*) values at steady state (10,000 year spin-up) throughout the period of the “bomb” spike (solid red line). Note that differences in pool ∆^14^C were small for the pre-bomb period but diverged rapidly in the 1960s. Similar single pool model curves were calculated during the parameter fitting procedure to estimate initial pool ∆^14^C for given *k* values

Given that the volume of archived soil limited further fractionation of samples to estimate the pool sizes and ∆^14^C from measurable C pools, the initial pool ∆^14^C was estimated during the fitting procedure. A one-pool model was run to steady state (12 000 year spin-up) to approximate a pool with given *k* (e.g. Figure 2). The initial fast pool ∆^14^C was estimated in an iterative procedure, using fitted *k1* values to compute pool ∆^14^C in 1958 and 1985. We assumed an initial slow pool ∆^14^C value of − 42.0 ‰ for every trial in 1958. For the second time window, the initial ∆^14^C values for fast and slow pools were calculated in each model run using the one-pool steady state model described above for the proposed *k1* and *k2* values of that run.

Carbon transit times, which describe the “ages of the particles at the time they leave the boundaries of a system; that is, the ages of the particles in the output flux” (Sierra et al. 2017) were calculated using the approach described by Metzler and Sierra (2018). Mean C inputs (Table 1) were used to build the vector ***I***, and fitted model parameters to build the matrix **A** for each time window. Computed transit time distributions have meaningful interpretations for the studied time windows because the values of ***I*** and **A** remain constant during these periods and the calculation of transit times does not assume C stocks to be at steady state (Manzoni et al. 2009; Sierra et al. 2021). In contrast, we did not compute the age distributions of C in the system because the stocks are not at steady state. While the computation of the transit time is only based on the assumption that new inputs will experience constant inputs and rates, the computation of the ages makes the critical assumption that the stocks experienced these constant inputs and rates for a sufficiently long-time (Manzoni et al. 2009; Sierra et al. 2021).

### Model performance and statistics

The parameters *k1*, *k2*, *a21*, and *slowProp* were estimated using Markov Chain Monte Carlo (*n* = 10 000) (Soetaert and Petzoldt 2010). The cost function relied on residuals between modeled and measured C stock and ^14^C signature for all years in the modeled time interval. Mean values and 99% confidence intervals were calculated using bootstrap resampling (Online Appendix Tables 1 and 2). A subset of 1000 accepted parameter sets were used to calculate variability of transit time, pool sizes, and times to steady state for each pool. Goodness of fit and parsimony was assessed using the Akaike information criterion (AIC).

## Results

### Measured C stocks and ∆^14^C

**Fig. 3:**
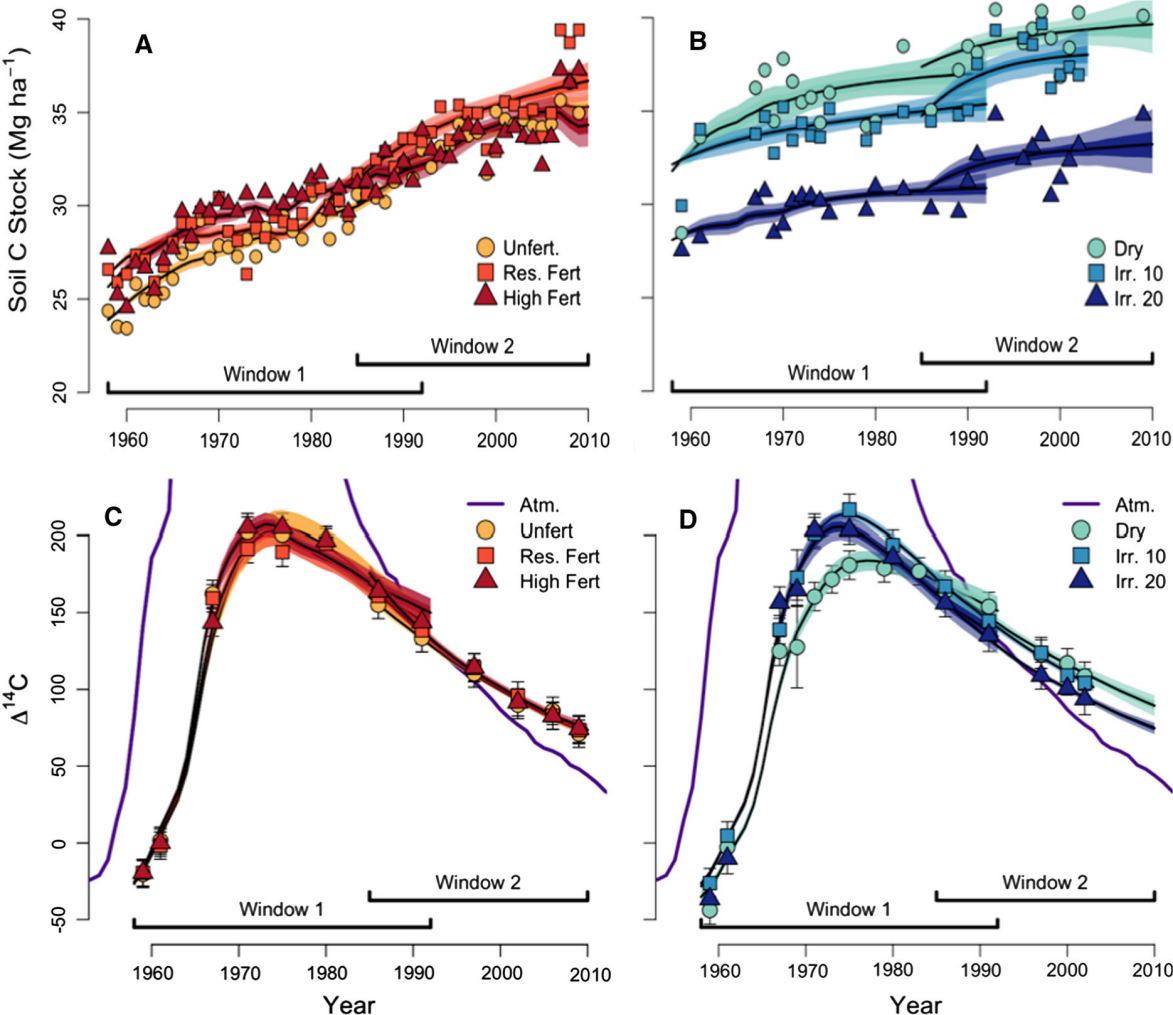
Observed and modeled SOC stocks for **A** fertilizer and **B** irrigation plots, respectively, with modeled time windows specified below. Data from 1959 to 2009 are presented originally in Schipper et al. (2013) and 2010 data are presented in Kelliher et al. (2012). Observed and modeled ∆^14^C for **C** fertilizer and **D** irrigation plots, respectively. In all plots, shaded areas contain model fits: black lines represent mean values, dark shaded areas are interquartile range model output values (25th-75th percentile), and lightly shaded areas represent 5th–95th percentiles of model output values (n = 1000)

Changes in soil C stocks were reported and discussed previously by Schipper et al. (2013) (Fig. 3 and Table 1). Fertilizer trials gained 0.20–0.22 Mg C ha^−1^ year^−1^ from 1959 to 2010, and irrigation trials gained 0.13—0.16 Mg C ha^−1^ year^−1^ from 1959 to 2002. In 1959, the first year for which data are available, Irr. 20 soils contained significantly less C (27.9 Mg C ha^−1^) than Dryland (31.4 Mg C ha^−1^) or Irr. 10 (32.1 Mg C ha^−1^), and this offset persisted for the duration of the experiment. Soil C stocks were also lower in 1959 (25.9–26.8 Mg C ha^−1^) in all fertilizer trial plots, which received irrigation at 15% soil moisture. A final sampling in 2009 (Kelliher et al. 2012) demonstrated that higher rates of accumulation persisted in High Fert and Res. Fert plots (0.27 to 0.37 Mg C ha^−1^ year^−1^ since 1995), were lower in Unfert and Irr. 20 (0.16 Mg C ha^−1^ year^−1^each), and smallest in Dry (0.08 Mg C ha^−1^ year^−1^).

Soil ∆^14^C changed markedly with time, but did not differ between fertilizer trials (Fig. 3c, d, Online Appendix Table 3). In 1959, all soils had negative ∆^14^C values although the atmosphere had ∆^14^C > 0. Dryland and Irr. 20 ∆^14^C were ~ 24‰ and ~ 17‰ lower than fertilizer treatments, respectively, and Dryland ∆^14^C remained significantly less than Irr. 10 or Irr. 20 until 1980, after which point ∆^14^C was higher until the end of the experiment (Fig. 3c, d).

### Model results

**Fig. 4:**
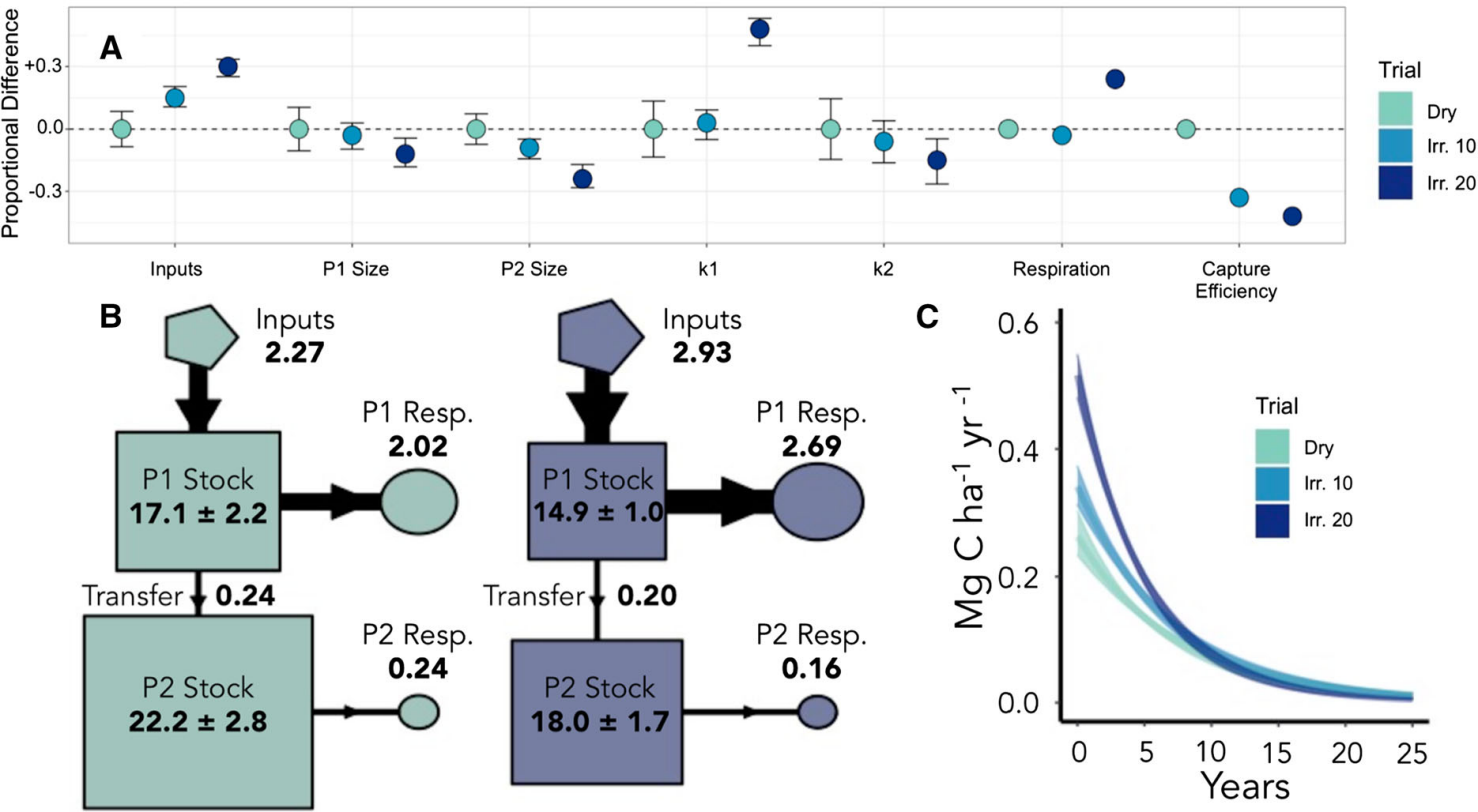
Modeled C dynamics for 1985–2010 modeling window in irrigation plots. **a** Relative properties and pool dynamics, normalized to Dry plots. Capture efficiency describes the amount of inputs stored annually (inputs/accumulation). **b** Model C flow diagrams (only Dry and Irr. 20 shown) for 2010. “P1” and “P2” refer to fast and slow pools, respectively. Flux boxes and pool boxes are internally proportionate. Numbers in bold represent the mass stock or flux in each step (Mg ha^−1^ year^−1^). **c** Mass-weighted transit time distributions of C input fluxes in 2009. The x-axis is truncated for visibility

**Fig. 5:**
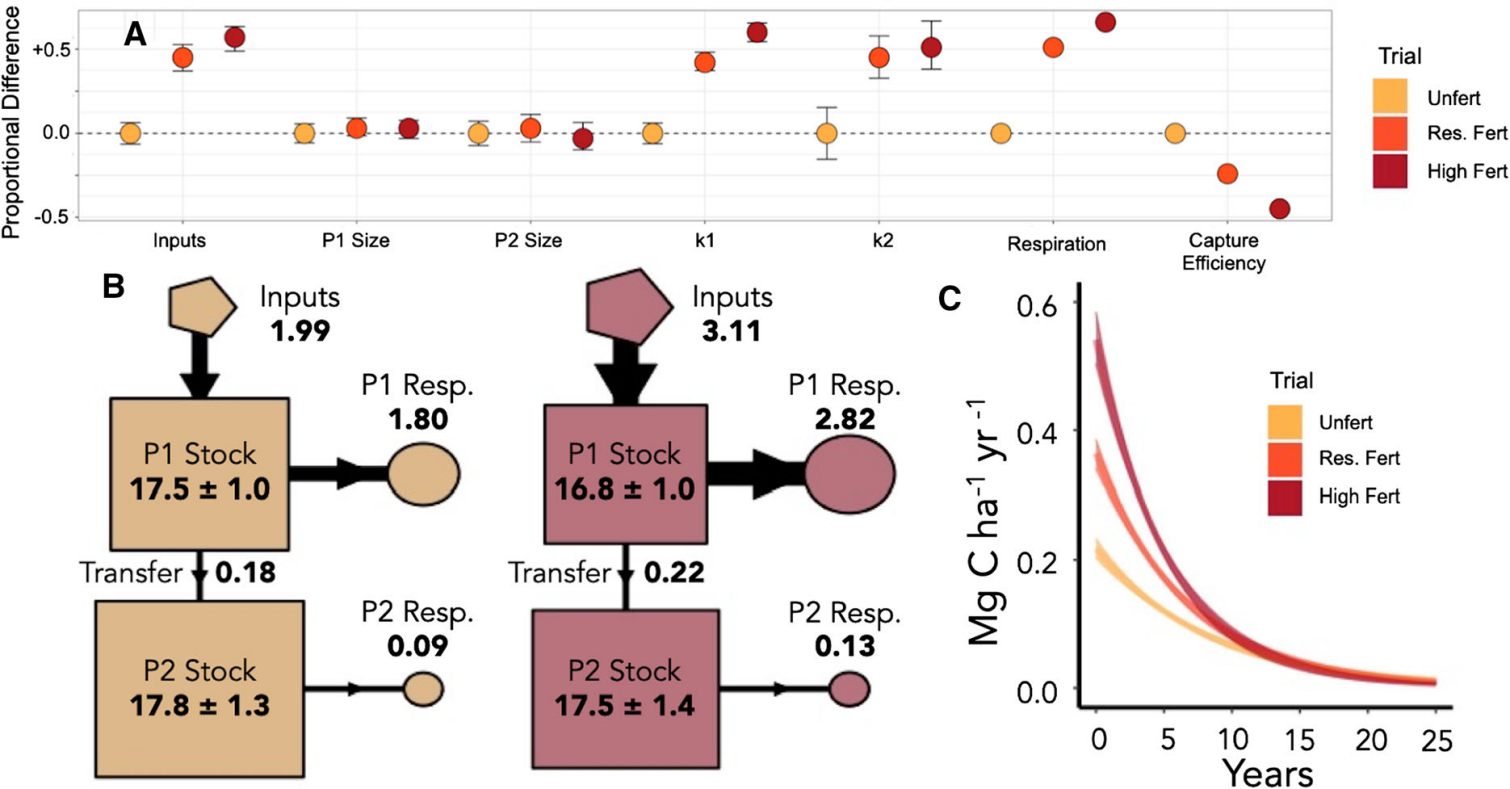
Modeled C dynamics for 1985–2010 modeling window in fertilizer plots. **a** Relative properties and pool dynamics, normalized to Unfert plots. Capture efficiency describes the amount of inputs stored annually (inputs/accumulation). **b** Flow diagrams (only Unfert and High Fert shown). Flux boxes and pool boxes are internally proportional. “P1” and “P2” refer to fast and slow pools, respectively. Bold values represent the mass stock or flux in each step (Mg ha^−1^ year^−1^). **c** Mass-weighted transit time distributions of C input fluxes in 2009. The x-axis is truncated for visibility

Model parameters are reported in Table 2, and summarized in Figs. 4 and 5; output on parameter covariance can be found in Online Appendix Fig. 6. Between modeled time windows, *k1* and *a21* values remained the same or decreased in all trials, and *k2* and *slowProp* decreased in all but Unfert where these parameters remained roughly constant. In other words, from the first to the second half of the record, there was a slowing of decay rates for both slow and fast pools, lower transfer rate from the fast to the slow pool, and a smaller proportion of C initially found in the slow pool. Following the assumption that the second window (1985–2010) better reflects the long-term management effects on C cycling and covers a time frame when soil ∆^14^C is less sensitive to changes related to the initial experimental setup, treatment comparisons will be discussed for the second window only (Table 2; see Online Appendix). In fertilizer trials, *k1* increased with inputs, and *k2* was greater in High Fert than Unfert or Res. Fert (Table 2, Fig. 5a). That is, High Fert had the most rapid fast and slow pool decay rates. Annual transfer from fast to slow pools (*a21*) was 1.3% in Res. Fert and High Fert in 2010, significantly (p < 0.01) greater than 1.0% in Unfert (Fig. 5b, only High Fert and Unfert shown). Unfert had the largest slow pool in 1985 (*slowProp*) of any fertilizer trial.

**Table 2:** Mean parameter values following Markov-Chain Monte Carlo analysis (n = 10,000)

Trial	Parameter	1958–1992 Mean (SD)	1985–2010 Mean (SD)	Trial	Parameter	1958–1992 Mean (SD)	1985–2010 Mean (SD)
Unfert	*k1*	0.14 (0.01)	0.11 (0.01)	Dry	*k1*	0.18 (0.02)	0.13 (0.02)
Res. fert		0.18 (0.02)	0.16 (0.01)	Irr. 10		0.17 (0.01)	0.14 (0.01)
High fert		0.29 (0.02)	0.18 (0.01)	Irr. 20		0.26 (0.02)	0.19 (0.01)
Unfert	*k2*	0.005 (0.003)	0.005 (0.001)	Dry	*k2*	0.02 (0.003)	0.011 (0.002)
Res. fert		0.009 (0.004)	0.007 (0.001)	Irr. 10		0.01 (0.002)	0.010 (0.001)
High fert		0.013 (0.002)	0.008 (0.001)	Irr. 20		0.015 (0.002)	0.009 (0.001)
Unfert	*alpha21*	0.15 (0.03)	0.09 (0.03)	Dry	*alpha21*	0.24 (0.04)	0.11 (0.06)
Res. fert		0.13 (0.03)	0.08 (0.03)	Irr. 10		0.13 (0.01)	0.06 (0.04)
High fert		0.15 (0.02)	0.07 (0.02)	Irr. 20		0.13 (0.02)	0.07 (0.03)
Unfert	*slowProp*	0.5 (0.04)	0.52 (0.02)	Dry	*slowProp*	0.79 (0.06)	0.59 (0.04)
Res. fert		0.55 (0.04)	0.51 (0.03)	Irr. 10		0.52 (0.02)	0.57 (0.03)
High fert		0.65 (0.03)	0.48 (0.03)	Irr. 20		0.64 (0.03)	0.55 (0.03)
Unfert	Median transit time	6.6 (0.4)	7.0 (0.2)	Dry	Median Transit Time	5.8 (0.3)	6.3 (0.4)
Res. fert		4.7 (0.2)	4.8 (0.1)	Irr. 10		5.0 (0.1)	5.6 (0.2)
High fert		4.3 (0.2)	4.3 (0.2)	Irr. 20		3.3 (0.2)	4.0 (0.2)

Decay rates *k1* and *k2*, while proportional slow pool size (*slowProp*) and *a21* decrease between model windows for most trials, leading to similar or increasing transit times (Table S1). Confidence intervals (99%) can be found in Online Appendix Tables 1 and 2. Median transit time is reported in years

In irrigation trials, Irr. 20 had the fastest *k1* and slowest *k2*, while Dryland and Irr. 10 did not differ (Table 2, Fig. 5a). Dryland had a slightly larger *a21* (Fig. 5a), transferring about 1.5% of total pool 1 stock to the slow pool annually compared to 0.8% in Irr. 10 and 1.4% in Irr. 20 (Fig. 5b, results for Dryland and Irr. 20 shown). The initial proportional slow pool stock (*slowProp*) was smallest in Irr. 20 in 1985.

Across treatments, fast pool inputs roughly matched outputs and stocks were stable by the end of the experiment in 2010 (Table 3; Online Appendix Fig. 4). At this time, fast pool C stocks varied significantly across the irrigation experiment (Irr. 10 > Irr. 20 > Dryland; Fig. 5a), but slow pool stocks for Irr. 10 and Irr. 20 were very similar and much smaller than Dryland, respectively (Fig. 4a). Res. Fert and High Fert had larger fast pool stocks, and tended to have smaller slow pool stocks, than Unfert.

**Table 3:** Mean transit times, and estimated time (from start of second time window) until fast pool and whole system C stocks approach steady state in top 0–7.5 cm for all trials

Trial	Mean transit time (years)	Years until 95% fast pool steady state stock	Years until 95% system steady state stocks
Unfert	25.7 ± 0.5	18 ± 1	395 ± 13
Res. fert	19.7 ± 0.3	14 ± 1	248 ± 9
High fert	14.7 ± 0.3	26 ± 1	233 ± 8
Dry	18.2 ± 0.4	9 ± 1	143 ± 8
Irr. 10	14.3 ± 0.5	11 ± 1	130 ± 9
Irr. 20	12.8 ± 0.3	5 ± 1	187 ± 9

Note that fast pools in all trials reach steady state by the end of the experiment. Steady state stock calculations assume constant inputs and environmental conditions (i.e. management continues indefinitely). Error represents 99% confidence interval from bootstrap resampling (R package rcompanion)

### Transit time

Median transit times of C inputs ranged from 3.3 to 7 years (Table 2). In Dryland and Unfert, half of the C added as inputs was lost through respiration within 6–7 years, while only 3.3 to 5 years was needed in more intensively managed plots. Median transit times increased slightly but significantly (p < 0.01) from the first to the second time window in all trials, reflecting slowing of decomposition rates (*k1* and *k2*; Table 2) and the accumulation of C in the slow pool (Online Appendix Fig. 4).

Transit time distributions (Figs. 4c and 5c) of inputs were highly skewed, and mean transit times (12–26 years; Table 3) are longer than the median transit times. The older (> 15 years) ‘tails’ of these distributions primarily reflect C losses from the slow pool, which contributes the majority of older C being lost from the system and where imbalances in inputs and losses are responsible for accumulation of C stocks in the models (Figs. 4b, 5b).

While fast C pools reached steady state by the end of the experiment (10–26 years, Table 3), slow pools were clearly still accumulating C. Assuming constant mean inputs for each treatment, we extrapolated that systems would reach 95% of steady state stock within 130–400 years, and steady state stocks would range from 41 to 53 Mg C ha^−1^, depending on treatment (compared to 32 to 39 Mg C ha^−1^ at the end of the experiment). Overall, irrigation treatments had similar estimated steady state C stocks, whereas greater fertilizer additions reduced estimated C stocks.

## Discussion

### Intensification increased inputs but not C accumulation

Data from the Winchmore trials indicate that soils are accumulating C at very similar rates, regardless of treatment (Schipper et al. 2013). The application of ^14^C constraints to modeling these trials illuminates three important points. First, as inputs increase, half of the added C is respired from the fast pools within ~ 4 years (median transit time) and the majority within 7–14 years (Figs. 4c, 5c; Tables 2, 3). Second, the overall rates of C accumulation (0.13–0.22 Mg C ha^−1^ year^−1^) are not sensitive to inputs and are controlled by the dynamics of the slow pool. Despite differences in inputs to the fast pool, transfer of C from the fast to the slow pool remained roughly constant. Thus, a smaller percentage of the total inputs in productive, intensive treatments were stored over subsequent decades, contributing to lower ‘capture efficiency’ (Figs. 4, 5; Table 1). Third, C continued to accumulate at constant rates despite larger stocks, reflected in slowing decay rates and transit times from the first to second modeling period (Table 2).

Even after 6 decades of management, the surface soils are still accumulating C and are far from steady state (Table 3). One potential explanation is that the soils are still accumulating C in recovery from topsoil scraping at the start of the experiment. If 2 cm of scraped soil with 5% C and a bulk density of 1.14 g cm^3^ were removed, this would amount to a loss of a maximum of ~ 11 Mg C ha^−1^. However, the newly exposed soil will not be starting with zero carbon; thus replacing lost C stocks would require far less total accumulation. In comparison, the soils gained 6–13 Mg C ha^−1^ over 60 years of management. While the controls on accumulation may be partly related to recovery of C lost in the initial disturbance involved in establishing border dykes, especially during the first modeling time window, it is unlikely that the C accumulated over the whole period is entirely from disturbance recovery.

### Key processes

Mechanisms underlying differences in C stabilization or decomposition rates with management include potential relationships to litter quality (Cordova et al. 2018), microbial biomass (e.g. Lange et al. 2015), the amount of ‘unsaturated’ available mineral surfaces (Dexter et al. 2008; Wiesmeier et al. 2015), and the formation of stable aggregates (Doetterl et al. 2012; Six et al. 2000; Tisdall and Oades 1982). Dominant mechanisms likely differed between trials, so we discuss each trial separately below.

#### Irrigation effects

In our study, fast pool decomposition rates increased with irrigation frequency, which may be related to observed greater earthworm and mesofauna activity in irrigated Winchmore plots (Fraser et al. 2012). High irrigation (Irr. 20) soils were kept continuously wet, at roughly 50% field capacity, while low irrigation (Irr. 10) soils approached permanent wilting point before each irrigation event. Thus Irr. 10 plots experienced more frequent severe drying and rewetting cycles than either of the other irrigation treatments, or the nutrient trials (all of which were irrigated when they had reached 15% soil moisture) (White et al. 2008). Slow-cycling C tends to be more vulnerable to decomposition under drying-rewetting conditions (Zhang et al. 2020), supported here by higher *k2* in Dryland and Irr. 10 plots compared to the perpetually moist Irr. 20 (Fig. 4a). These results support the general observation that seasonally dry grasslands under more humid conditions tend to have less SOM than drier soils (WRB 2015).

Despite accumulating C at similar rates, bulk C stocks for the top 7.5 cm in the Irr. 20 plot are markedly (and Irr. 10 marginally) lower than all other treatments (Fig. 3a, b). This reduced C storage extended to greater depths; at the end of the experiment Irr. 20 plots stored 33% less C than Dryland plots in the top 1 m of soil (Kelliher et al. 2012). As all plots were initially subjected to the same topsoil removal, Schipper et al. (2013) inferred a rapid loss of C in the decade following initial conversion of the Winchmore trials to irrigation management in 1948 prior to sampling in 1959. This is supported by a study of paired irrigated and unirrigated pastures in the same region that demonstrated on average 1.4 Mg ha^−1^ lower C stocks in topsoils (0–10 cm) irrigated by center-pivot within 8–15 years of beginning irrigation treatment (Mudge et al. 2017). In the Winchmore trials, despite approximately equal inputs, the Dryland soils had more and generally older C in 1959 than Unfert (which was irrigated at 15%), suggesting a loss of younger C in wetter soils (Fig. 3). In the pre-1959 period, a lack of samples and small expected differences in ∆^14^C between fast and slow pools do not allow us to further constrain potential initial C losses. However, these results suggest that adoption of irrigation can lead to rapid reductions in C stocks, though this did not affect subsequent rates of C accumulation across treatments.

#### Fertilizer effects

Stimulated fast pool decomposition with higher fertilizer inputs coincide with increases in litter quality (Cordova et al. 2018). Carbon to nitrogen (C:N) ratios decreased from 22.3 in unfertilized plots to 16.2 in highly fertilized plots (Online Appendix Fig. 2). In addition, microbial biomass increased with P fertilizer rate (Wakelin et al. 2017), though there was no observed change in microbial community composition (Condron et al. 2006). Thus higher decomposition rates reflect increased microbial activity, rather than community changes.

Decomposition rates of the slow pool increased with fertilizer addition and offset the approximately 20% greater transfer to the slow pool in high fertilizer treatments (Fig. 5b). One explanation for faster slow pool decomposition rates would be ‘priming’—losses of stable SOM that accompany greater fresh C inputs (Kuzyakov and Blagodatskaya 2015), or through relief of C limitation in fertilized soils (Demoling et al. 2007; Soong et al. 2019). However, large priming losses associated with increased nutrient and substrate inputs were not observed during the early stages of the experiments, as C stocks did not differ with nutrient addition. Therefore, either priming losses were small, or lost C was rapidly replaced with newly fixed C. The similarity of ∆^14^C in 1959 and onward across fertilizer treatments does not suggest a large priming effect. While increased inputs led to increased overall decomposition rates in fertilizer trials, they did not result in large losses of pre-treatment (pre-bomb) soil C stocks.

### Model implications and limitations

#### Model structure

Linear dynamical models predict that increased inputs under constant environmental conditions will increase soil C stocks to new steady state (West and Six 2007) because the first-order decomposition rate (*k*) is constant, and thus C pool sizes will increase until losses (k*C) equal inputs. Such linear models do allow for modification of decomposition rates, for example, as a function of soil moisture, a phenomenon that is well-documented (Sierra et al. 2015). However, in the P fertilization trials, where all plots were irrigated at 15% soil moisture, decomposition rates increased with P addition in both fast and slow pools. The explanation for increased *k*’s in these plots cannot be environmental factors alone, but require additionally that decomposition rates increase with inputs, likely through processes linked to changes in soil microbial activity, e.g. nutrient and C availability (Soong et al. 2019).

The use of models allows quantitative comparisons and demonstrates the differences in decomposition rates among the treatments at the Winchmore site. However, while we give 99% confidence intervals for the parameters fit to C and ∆^14^C data with our two-pool model, there are uncertainties about the overall accuracy of rates we obtained because they are specific to the model structure applied (Sierra et al. 2015). The uncertainties associated with ^114^C modeling have been discussed in depth previously (Sanderman et al. 2016; Baisden et al. 2002; Baisden and Canessa 2013). For example, the two-pool structure of our model excludes a commonly used “passive” pool (Parton et al. 1987; Baisden and Canessa 2013), with decomposition rates typically << 0.001 year^−1^. Adding such a ‘passive’ pool did not improve our ability to fit the data (AIC: 7.7 ± 1.3 for two pools vs. 13.8 ± 1.4 for three pools) and would generally increase the cycling rates of C in the slow pool, with little relative change in overall cycling rates across the treatments in the long-term experiment. Including another pool requires additional assumptions and/or fitting of up to three additional parameters while compounding the uncertainty of initial pool sizes and ∆^14^C values. In the future, model structures that include microbial processes such as recycling of C could be tested against the Winchmore data set to determine whether they could provide a better and parsimonious fit over the whole time period.

#### Initial conditions and changes over time

Additional sources of uncertainty in our model result from unknown initial conditions at the start of treatment in 1948 and changing rates of C cycling over time. First, a lack of data on initial conditions in particular limited our ability to understand the degree to which irrigation and fertilization may have led to substantial C losses in the first decade of treatment. Second, we were unable to fit the complete time series with a single set of parameters, likely as a result of the evolving response to management and recovery from topsoil scraping. Finally, the lack of a true control (no fertilizer and no irrigation) limits the ability to differentiate among potential mechanisms for differences in dynamics among treatments.

### Implications for grasslands

We observed faster SOM decomposition in the most intensively managed pastures, resulting in transit times up to 50% faster than respective control plots (Table 2). Such accelerated cycling has important implications for grazed grasslands under common management practices. The relatively slow decomposition rate of the slow pool in unfertilized soil indicates that a greater proportion of the reduced inputs were stabilized. Treatment-related increases in C inputs mostly remain in the system for 1–15 years (Figs. 4c, 5c), while a small fraction (approx. 0.18–0.24 Mg C ha^−1^ year^−1^) of inputs are stored, regardless of treatment, on decadal time scales. Slowing of decomposition rates between the two time windows allows continued C accumulation at near-constant rates over the 60-year experiment. We cannot explain this observation, but only speculate that C stabilization shifts over time from initial disturbance recovery to mechanisms that operate on longer timescales.

Disturbances, such as overgrazing, tillage, erosion, or conversion to cropland, that reduce C inputs and alter soil structure in ways that can accelerate decomposition generally reduce C storage in grasslands (Soussana et al. 2004; Stewart et al. 2007; Minasny et al. 2017). We argue that, in our study, lower C stocks in irrigated treatments could reflect rapid C loss associated with increased water application and related disturbance (Kelliher et al. 2012; Mudge et al. 2017). Stocks can be recovered, but may be particularly vulnerable to loss with renewed disturbance. Reducing disturbances that can accelerate decomposition rates, including those potentially associated with irrigation, is thus important for storing C in grasslands.

Many recommendations for sequestering SOM in degraded or low-productivity soils implicitly assume that greater productivity will translate into increased long-term C storage (Conant et al. 2017). In our study, we find that increased C inputs are rapidly respired and do not necessarily increase long-term C storage, consistent with recent literature (Cambell et al. 1991; Stewart et al. 2007; Sanderman et al. 2017). Thus, decreases in soil C capture efficiency in the treatments investigated represent a potential obstacle to soil C storage as a tool for mitigating climate change once comparably small fast-cycling soil C pools have saturated.

Carbon accumulated consistently in the top 7.5 cm of fertilizer trials at rates of 8.0 ∓ 2.6 per mille year^−1^, and irrigation trials at rates of 3.7 ∓ 2.5 per mille year^−1^ (mean annual increase of 0.80% and 3.7% of 1958 C stocks, respectively) over the course of the experiment. High rates found in the fertilizer trails highlight the potential for semi-arid grasslands to sequester large amounts of C (Trost et al. 2013) and to maintain C sequestration at rates in line with the global goal of “4 per mille” annual C stock increase (Minasny et al. 2017). However, disturbance in Winchmore soils due to border-dyke construction and irrigation was likely responsible for some C loss, and initially high accumulation rates could partly reflect disturbance recovery. While C rapidly accumulates following conversion to managed pasture here and elsewhere (Machmuller et al. 2015), sustained rates of accumulation in the slow pool highlight the importance of “dynamic stability” in C sequestration efforts (Dynarksi et al. 2020). While it may not always store more C, intensification can contribute to other negative outcomes (e.g. non-CO_2_ greenhouse gasses, acidification, erosion) that may offset gains from C storage in greenhouse gas equivalents rates. Increasing grassland productivity through these management practices will increase C stocks in the short term (years to decades), but lasting effects will require sustained effort.

## Conclusion

Soil archiving allowed for a unique application of ^14^C to model long-term effects on C storage in changing, managed systems. Generally, increased organic matter inputs associated with management intensification were largely matched by increased decomposition losses from pools with fast cycling rates, such that stabilized C did not increase in step with inputs and additional C only remained in the system for 5–15 years. While high fertilizer application enhanced long-term microbial decomposition in both fast and slow C pools, we conclude that there was no large initial priming loss of old C due to fertilizer application and subsequent productivity increases. Over 60 years of management, similar rates of C accumulation were observed within experiments, which highlight an overall similarity between treatments receiving both fertilizer and irrigation, and may indicate rate-limiting controls on C storage. The cumulative effects of management observed here must be considered when adopting strategies to optimize productivity, soil health, and long-term SOM sequestration. Overall, our findings imply that grassland management to increase productivity does not necessarily increase rates of SOM sequestration.

## Supplementary Information

Below is the link to the electronic supplementary material.

Supplementary file1 (DOCX 2157 kb)

## Data Availability

Data are available with code via git repository https://github.com/ShaneStoner/Winchmore14C for review. Dataset will be published with a DOI that can be cited in the publication.

## Code availability

Code can be found in the above git repository.
