# Supplementary material for: Soil organic matter turnover rates increase to match increased inputs in grazed grasslands
Source: Biogeochemistry. 2021 Aug 27;156(1):145–60. doi: 10.1007/s10533-021-00838-z (PMC8550221; doi:10.1007/s10533-021-00838-z)
Supplement: Supplementary file 1 — Supplementary file1 (DOCX 2157 kb) [file 10533_2021_838_MOESM1_ESM.docx]

**Supplemental Figures**


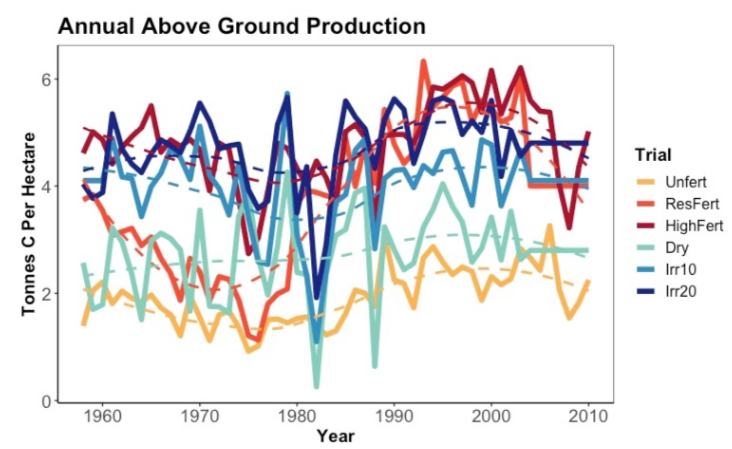


**Appendix figure 1:** Annual input data after *θ* factor adjustments (Table 1) (aboveground production only, assuming 40% of DM production as C content (Saggar and Hedley, 2001). Where no annual data was available, mean values were used. Mean annual root production data from Scott et al., (2012) (Table 2). Dashed lines represent data smoothing using *loess* regression.

 
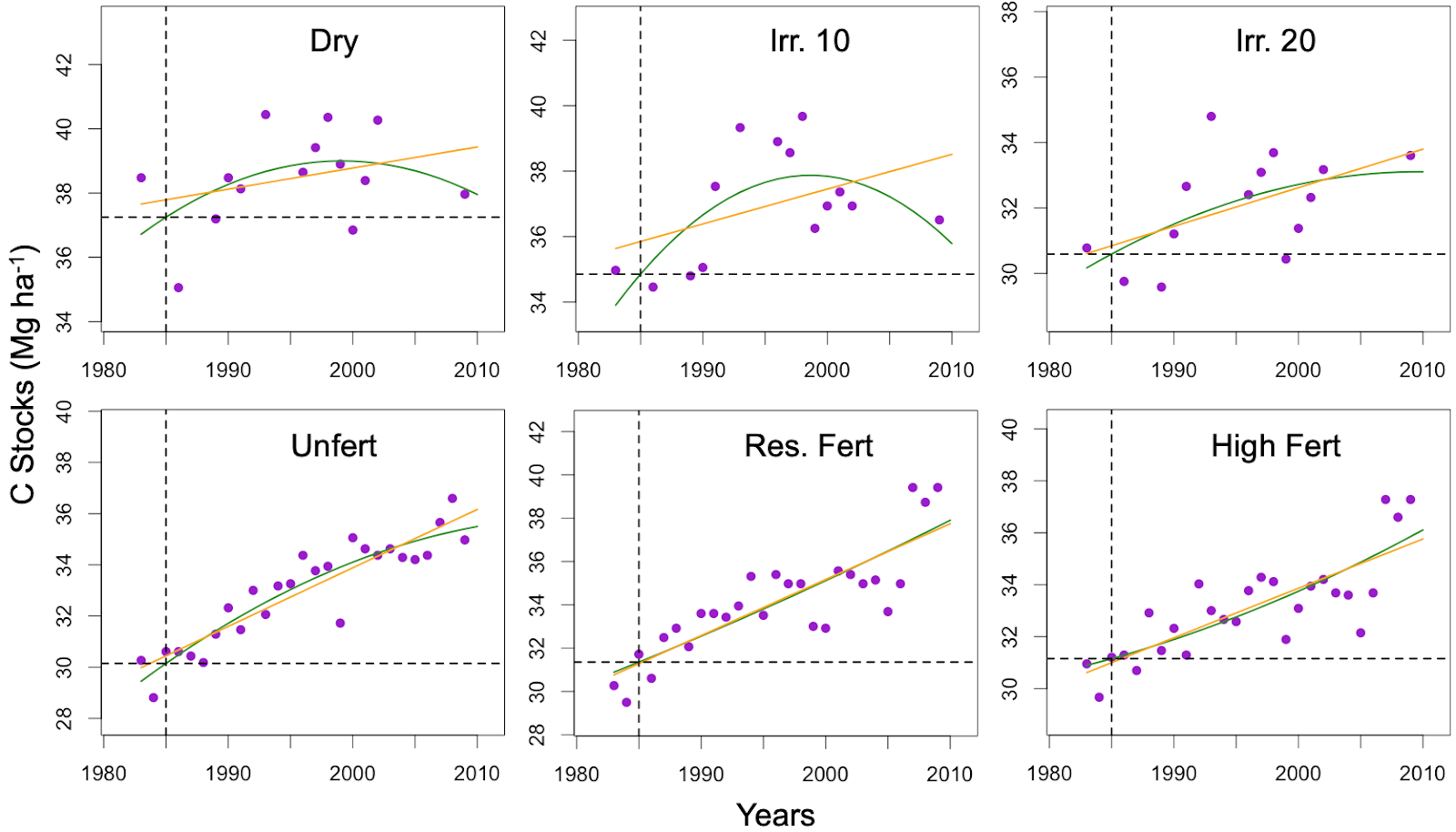


**Appendix figure 2:** Regression plots used to estimate initial C content in trials (only window 2 shown). Purple points are soil C stock data. Yellow lines represent linear regression fits. Green lines represent polynomial regression fits, which were used for all trials to select initial C stock values (indicated by crossing of dashed lines).

 
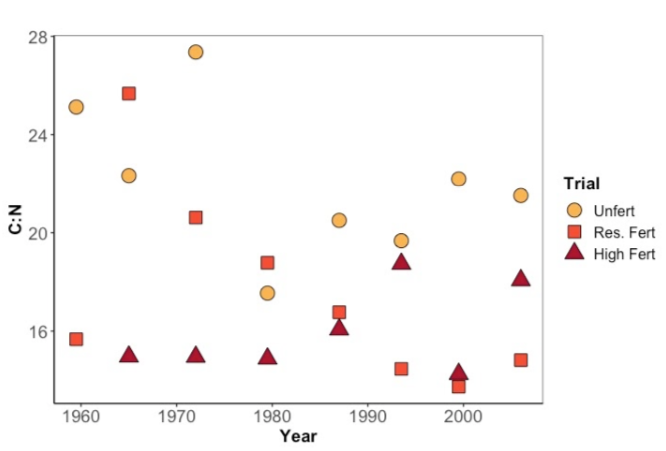


**Appendix figure 3:** Aboveground litter C:N for fertilizer trials, assuming a C content of 40% (Saggar and Hedley, 2001). Note that no data is available for High Fert in 1959.


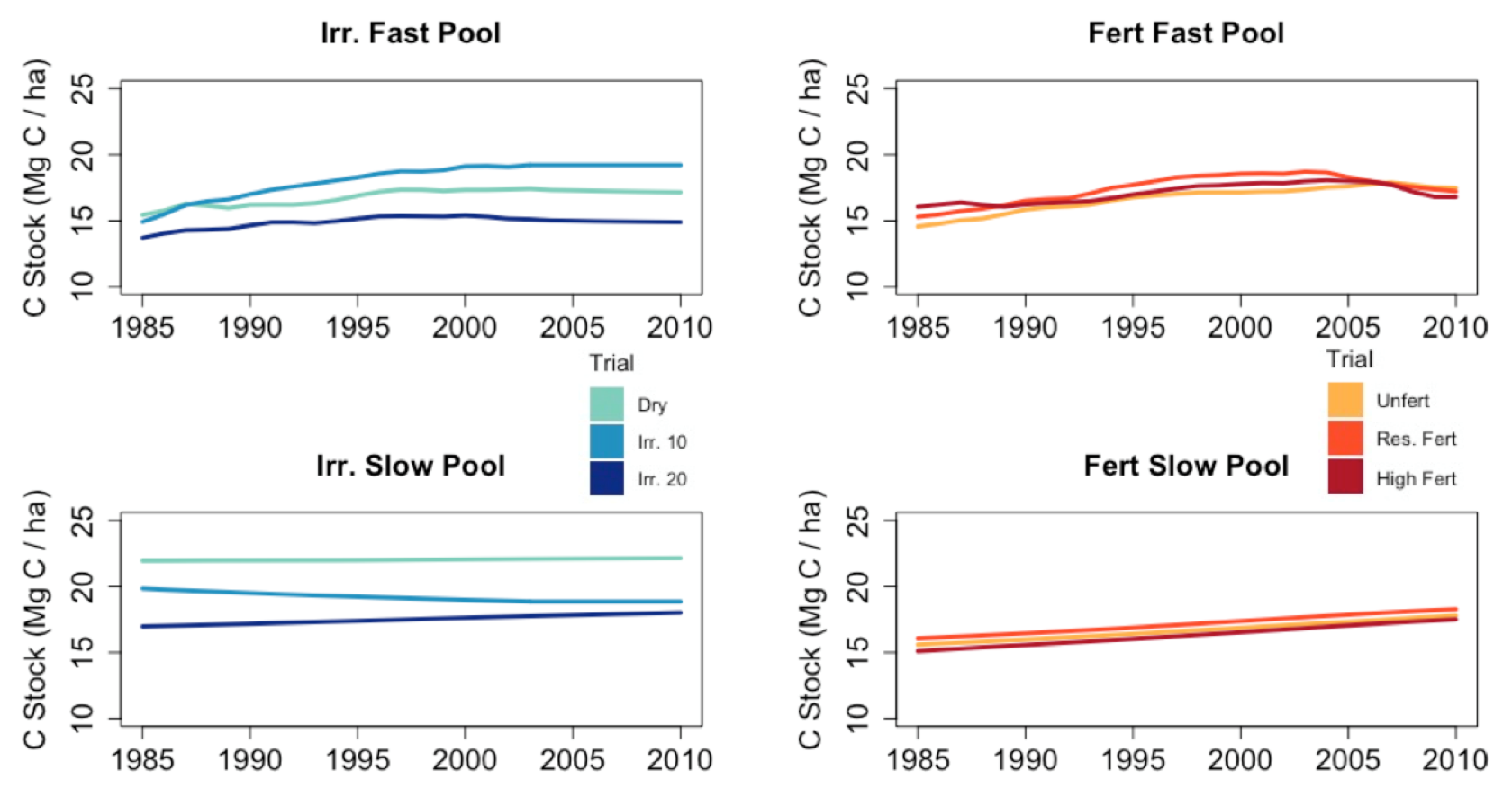


**Appendix figure 4** Mean modeled C pool sizes (Mg C ha^-1^) over time for each treatment (1985-2010) following Markov-Chain Monte Carlo parameter estimation. Long-term gains in C stock are generally in the slow pool (P2), and fast pool (P1) tend to reach steady state by 2010 (Table 3).


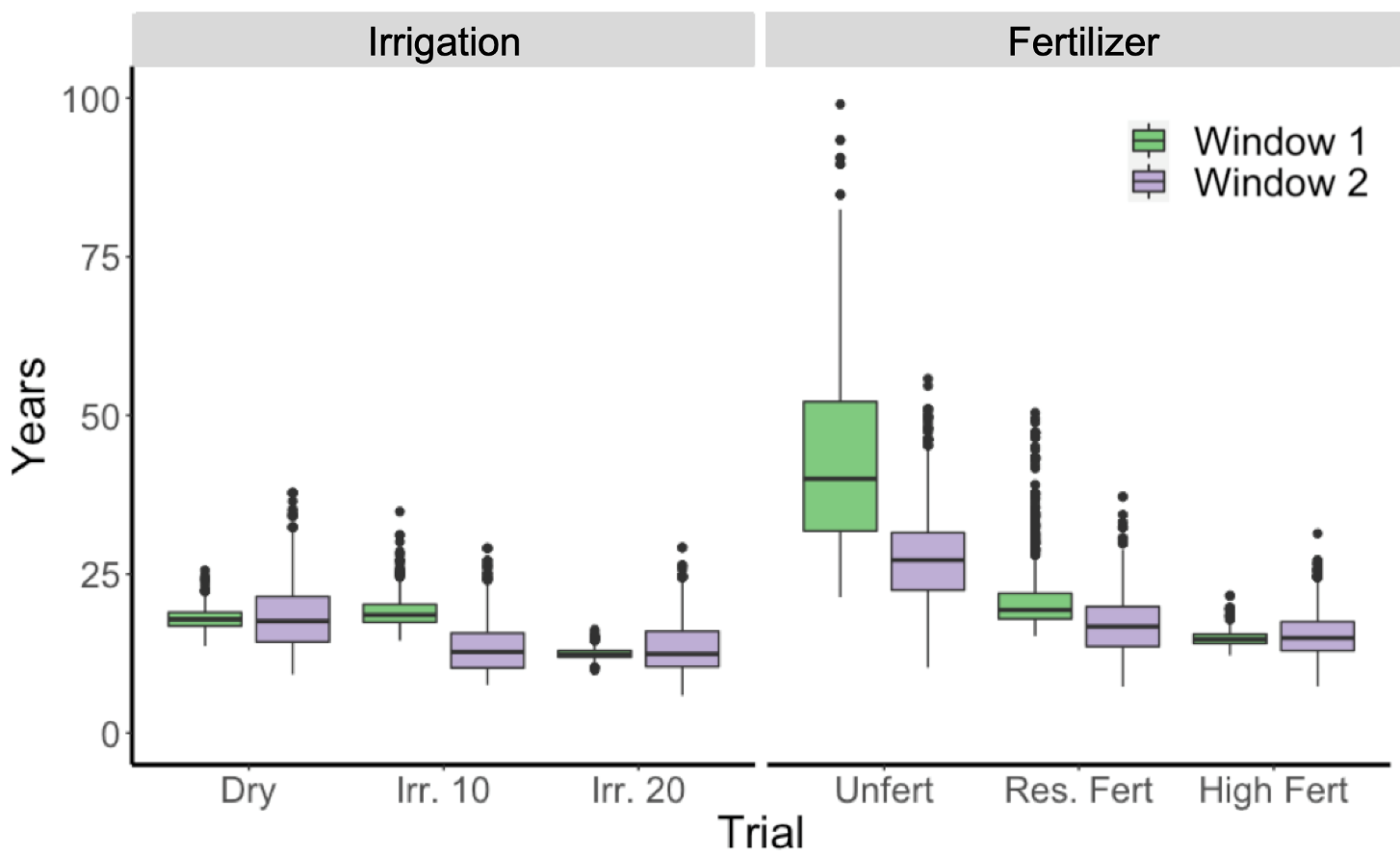


**Appendix figure 5:** Mean transit time calculated with parameters and inputs from each windows 1 and (1958-1992 and 1985-2010, respectively).


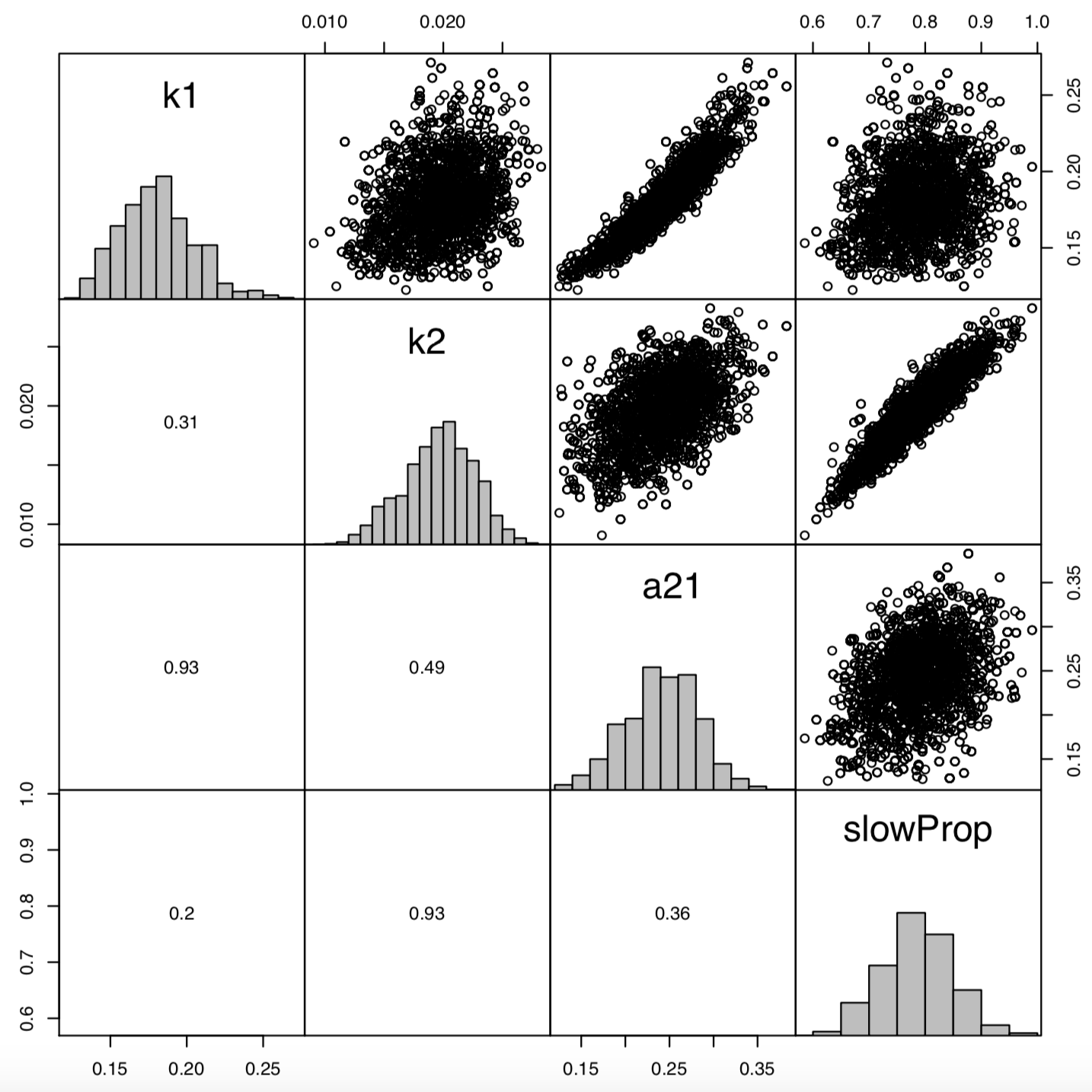


**Appendix figure 6:** Example covariance diagram of model parameters (Dryland, 1958-1992).


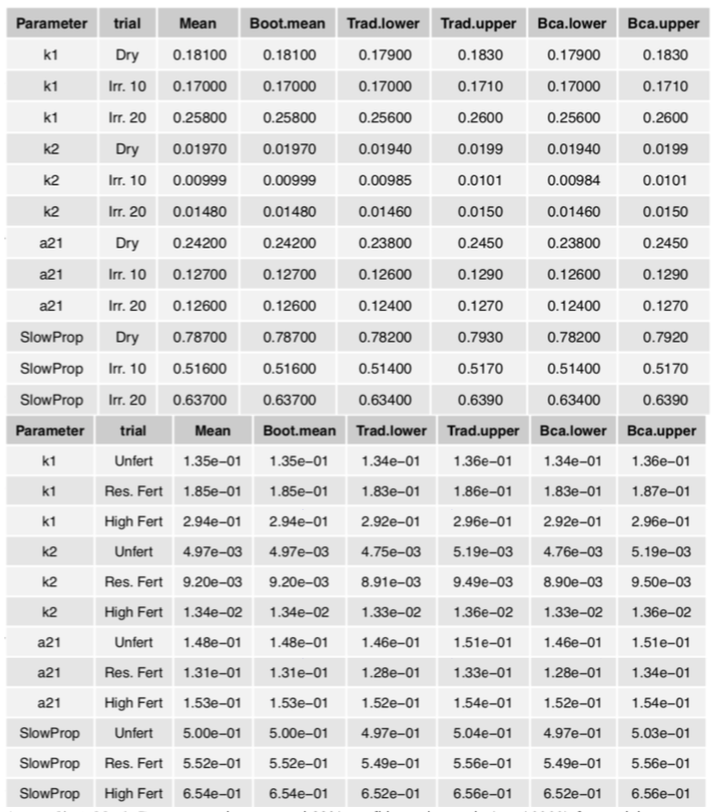


**Appendix table 1:** Bootstrapped means and 99% confidence intervals (n = 10000) for model parameters from 1958-1992 time window estimated using Markov-Chain Monte Carlo estimation. “Trad.lower” and “Trad.upper” refer to traditional confidence intervals for group means using Student’s *t*-distribution. “Bca.lower” and “Bca.upper” refer to bias corrected and accelerated confidence intervals (Davison et al., 1997).


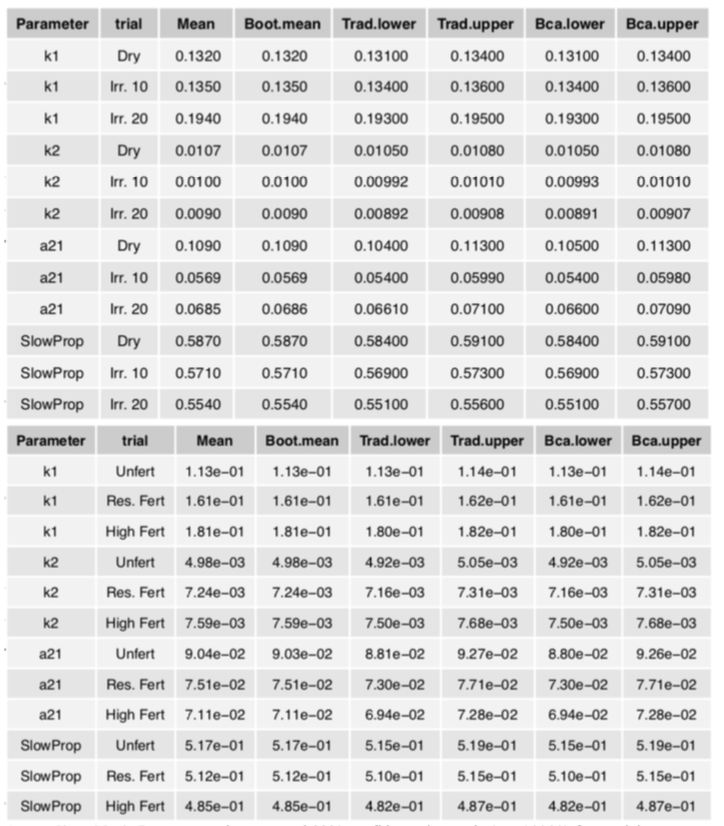


**Appendix table 2:** Bootstrapped means and 99% confidence intervals (n = 10000) for model parameters from 1985-2010 time window estimated using Markov-Chain Monte Carlo parameter estimation. “Trad.lower” and “Trad.upper” refer to traditional confidence intervals for group means using Student’s *t*-distribution. “Bca.lower” and “Bca.upper” refer to bias corrected and accelerated confidence intervals (Davison et al., 1997).

| AMS  Lab Code | Lab  Sample ID | Treatment | Year | Fm | Fm Error | ∆^14^C  (‰) | ∆^14^C error  (‰) |
| --- | --- | --- | --- | --- | --- | --- | --- |
| P | 19061 | Control | 1959 | 0.9807 | 0.0015 | -20.4 | 1.5 |
| P | 19062 | Control | 1961 | 1.0029 | 0.0015 | 1.6 | 1.5 |
| P | 19063 | Control | 1967 | 1.1646 | 0.0016 | 162.2 | 1.6 |
| P | 19064 | Control | 1971 | 1.2056 | 0.0018 | 202.5 | 1.8 |
| P | 19065 | Control | 1975 | 1.2042 | 0.0019 | 200.6 | 1.9 |
| P | 19066 | Control | 1980 | 1.2142 | 0.0017 | 209.8 | 1.7 |
| P | 19067 | Control | 1986 | 1.1600 | 0.0016 | 155.0 | 1.6 |
| P | 19068 | Control | 1991 | 1.1389 | 0.0017 | 133.3 | 1.7 |
| P | 19069 | Control | 1997 | 1.1167 | 0.0016 | 110.4 | 1.6 |
| P | 19070 | Control | 2002 | 1.0968 | 0.0017 | 89.9 | 1.7 |
| P | 19071 | Control | 2006 | 1.0934 | 0.0017 | 86.0 | 1.7 |
| P | 19072 | Control | 2009 | 1.0788 | 0.0016 | 71.1 | 1.6 |
|  |  |  |  |  |  |  |  |
| P | 19073 | Res. Fert | 1959 | 0.9816 | 0.0014 | -19.5 | 1.4 |
| P | 19074 | Res. Fert | 1961 | 0.9996 | 0.0016 | -1.7 | 1.6 |
| P | 19075 | Res. Fert | 1967 | 1.1617 | 0.0017 | 159.3 | 1.7 |
| P | 19076 | Res. Fert | 1971 | 1.1944 | 0.0016 | 191.4 | 1.6 |
| P | 19077 | Res. Fert | 1975 | 1.1930 | 0.0017 | 189.4 | 1.8 |
| P | 19078 | Res. Fert | 1980 | 1.1994 | 0.0016 | 195.1 | 1.6 |
| P | 19079 | Res. Fert | 1986 | 1.1659 | 0.0017 | 160.8 | 1.7 |
| P | 19080 | Res. Fert | 1991 | 1.1440 | 0.0018 | 138.3 | 1.8 |
| P | 19081 | Res. Fert | 1997 | 1.1204 | 0.0016 | 114.0 | 1.6 |
| P | 19082 | Res. Fert | 2002 | 1.1029 | 0.0016 | 96.0 | 1.6 |
| P | 19083 | Res. Fert | 2006 | 1.0903 | 0.0015 | 82.9 | 1.5 |
| P | 19084 | Res. Fert | 2009 | 1.0811 | 0.0015 | 73.4 | 1.5 |
|  |  |  |  |  |  |  |  |
| P | 19085 | High Fert | 1959 | 0.9817 | 0.0015 | -19.4 | 1.5 |
| P | 19086 | High Fert | 1961 | 1.0015 | 0.0016 | 0.2 | 1.6 |
| P | 19087 | High Fert | 1967 | 1.1458 | 0.0017 | 143.4 | 1.7 |
| P | 19088 | High Fert | 1971 | 1.2081 | 0.0017 | 205.0 | 1.7 |
| P | 19089 | High Fert | 1975 | 1.2085 | 0.0021 | 204.9 | 2.1 |
| P | 19090 | High Fert | 1980 | 1.2010 | 0.0017 | 196.6 | 1.7 |
| P | 19091 | High Fert | 1986 | 1.1689 | 0.0018 | 163.8 | 1.8 |
| P | 19092 | High Fert | 1991 | 1.1494 | 0.0016 | 143.7 | 1.6 |
| P | 19093 | High Fert | 1997 | 1.1208 | 0.0018 | 114.4 | 1.8 |
| P | 19094 | High Fert | 2002 | 1.0985 | 0.0017 | 91.6 | 1.7 |
| P | 19095 | High Fert | 2006 | 1.0900 | 0.0016 | 82.6 | 1.6 |
| P | 19096 | High Fert | 2009 | 1.0820 | 0.0016 | 74.3 | 1.6 |

**Appendix table 3**: Radiocarbon values for fertilizer trial archive samples as reported by laboratories. ∆^14^C are decay-corrected for date of collection. Data from Max Planck Institute for Biogeochemistry (AMS lab code P) were collected in 2018.

| AMS Lab Code | Lab  Sample ID | Treatment | Year | Fm | Fm Error | ∆^14^C  (‰) | ∆^14^C error  (‰) |
| --- | --- | --- | --- | --- | --- | --- | --- |
| NZA | 39414 | Dry | 1959 | 0.9633 | 0.0018 | -43.9 | 1.8 |
| NZA | 39415 | Dry | 1961 | 1.0046 | 0.0019 | -2.9 | 1.9 |
| NZA | 39426 | Dry | 1967 | 1.1333 | 0.0020 | 124.9 | 2.0 |
| NZA | 39428 | Dry | 1971 | 1.1692 | 0.0020 | 160.4 | 2.0 |
| P | 19261 | Dry | 1973 | 1.1749 | 0.0017 | 171.6 | 1.7 |
| NZA | 39427 | Dry | 1975 | 1.1898 | 0.0021 | 180.9 | 2.0 |
| P | 19262 | Dry | 1979 | 1.1829 | 0.0022 | 178.8 | 2.2 |
| NZA | 39429 | Dry | 1980 | 1.1669 | 0.0020 | 158.2 | 2.0 |
| P | 19263 | Dry | 1983 | 1.1816 | 0.0022 | 176.9 | 2.2 |
| NZA | 39425 | Dry | 1986 | 1.1726 | 0.0021 | 163.9 | 2.0 |
| NZA | 39424 | Dry | 1991 | 1.1626 | 0.0020 | 153.9 | 2.0 |
| NZA | 39423 | Dry | 1997 | 1.1313 | 0.0020 | 122.9 | 2.0 |
| NZA | 39422 | Dry | 2002 | 1.1169 | 0.0020 | 108.6 | 2.0 |
|  |  |  |  |  |  |  |  |
| NZA | 39606 | Irr. 10 | 1959 | 0.9811 | 0.0023 | -26.2 | 2.3 |
| NZA | 39416 | Irr. 10 | 1961 | 1.0123 | 0.0019 | 4.8 | 1.8 |
| NZA | 39419 | Irr. 10 | 1967 | 1.1473 | 0.0020 | 138.8 | 2.0 |
| NZA | 39418 | Irr. 10 | 1971 | 1.2105 | 0.0026 | 201.5 | 2.6 |
| NZA | 39607 | Irr. 10 | 1975 | 1.2254 | 0.0027 | 216.3 | 2.7 |
| NZA | 39417 | Irr. 10 | 1980 | 1.2029 | 0.0021 | 193.9 | 2.0 |
| NZA | 39720 | Irr. 10 | 1986 | 1.1759 | 0.0028 | 167.1 | 2.8 |
| NZA | 39420 | Irr. 10 | 1991 | 1.1529 | 0.0020 | 144.3 | 2.0 |
| NZA | 39721 | Irr. 10 | 1997 | 1.1323 | 0.0027 | 123.8 | 2.7 |
| NZA | 39421 | Irr. 10 | 2002 | 1.1126 | 0.0020 | 104.3 | 1.9 |
|  |  |  |  |  |  |  |  |
| NZA | 39907 | Irr. 20 | 1959 | 0.9706 | 0.0015 | -36.6 | 1.5 |
| NZA | 39499 | Irr. 20 | 1961 | 0.9974 | 0.0027 | -10.1 | 2.7 |
| NZA | 39500 | Irr. 20 | 1967 | 1.1652 | 0.0030 | 156.5 | 3.0 |
| NZA | 39501 | Irr. 20 | 1971 | 1.2123 | 0.0031 | 203.3 | 3.1 |
| NZA | 39908 | Irr. 20 | 1975 | 1.2124 | 0.0017 | 203.3 | 1.7 |
| NZA | 39502 | Irr. 20 | 1980 | 1.1949 | 0.0031 | 185.9 | 3.1 |
| NZA | 39909 | Irr. 20 | 1986 | 1.1648 | 0.0016 | 156.1 | 1.6 |
| NZA | 39503 | Irr. 20 | 1991 | 1.1437 | 0.0033 | 135.2 | 3.3 |
| NZA | 39910 | Irr. 20 | 1997 | 1.1174 | 0.0015 | 109.0 | 1.5 |
| NZA | 39504 | Irr. 20 | 2002 | 1.1019 | 0.0029 | 93.7 | 2.9 |
|  |  |  |  |  |  |  |  |
|  |  |  |  |  |  |  |  |

**Appendix table 4**: Radiocarbon values for irrigation trial archive samples as reported by laboratories. ∆^14^C are decay-corrected for date of collection. Data from GNS Science Rafter lab (AMS lab code NZA) were collected in 2012. Data from Max Planck Institute for Biogeochemistry (AMS lab code P) were collected in 2018.
